# Images of bronchiectasis in thoracic surgery

**DOI:** 10.11604/pamj.2015.22.20.7085

**Published:** 2015-09-09

**Authors:** Grégoire Kouakou Ayegnon, Christophe Gueu Ménéas

**Affiliations:** 1 Department of Cardio Vascular and Thoracic Diseases, Bouaké University Teaching Hospital, Bouaké, Côte d'Ivoire

**Keywords:** Bronchietasis, tuberculosis sequelae, lobarbronchiectasis

## Image in medicine

The bronchial dilations also called bronchiectasis are permanent and irreversible increase in the bronchial tubes. They can be extended or localized especially in pulmonary tuberculosis sequelae. This affection is serious, because it is at the origin of an embarrassing obstructive pulmonary disease, leading to social discomfort and preferentially in Côte d'Ivoire, it affects young subjects between 30 and 40 years old and former tuberculous. The place of surgery is still debated. Mr Coulibaly is 30 years old, hospitalized in the Thoracic Surgery Department of BouakeTeaching Hospital for pulmonary tuberculosis sequelae type symptomatic left lower bronchiectasis lobe and well localized (A). After a satisfactory preoperative evaluation, we performed a left lower lobectomy on this patient. Transection in the third world of the bronchial lower lobe resected reveals multiple tubular dilations with thickened wall containing purulent secretions (B). The specimen was sent to the pathology laboratory for confirmation of tuberculosis sequelae.

**Figure 1:**
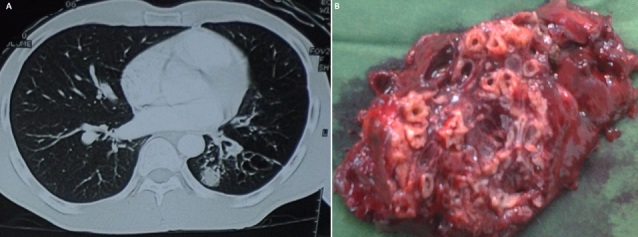
(A)thoracic Scanshowing the localized left lower lobarbronchiectasis; (B): bronchiectasis of lower left lobar surrinfected, viewed after transection of the left lower lobectomy of lung

